# The Emerging Role of Long Non-Coding RNAs in Esophageal Cancer: Functions in Tumorigenesis and Clinical Implications

**DOI:** 10.3389/fphar.2022.885075

**Published:** 2022-05-13

**Authors:** Yali Han, Guo Zhao, Xinhang Shi, Yushan Wang, Xin Wen, Lu Zhang, Xiangqian Guo

**Affiliations:** ^1^ Departments of Physiology, Institute of Biomedical Informatics, Bioinformatics Center, Henan Provincial Engineering Center for Tumor Molecular Medicine, School of Basic Medical Sciences, Academy for Advanced Interdisciplinary Studies, Henan University, Kaifeng, China; ^2^ Department of Preventive Medicine, Institute of Biomedical Informatics, Bioinformatics Center, Henan Provincial Engineering Center for Tumor Molecular Medicine, School of Basic Medical Sciences, Academy for Advanced Interdisciplinary Studies, Henan University, Kaifeng, China

**Keywords:** long non-coding RNAs, esophageal cancer, biological function, clinical application, cancer therapy

## Abstract

Esophageal cancer (EC) is one of the most common malignancies of digestive tracts with poor five-year survival rate. Hence, it is very significant to further investigate the occurrence and development mechanism of esophageal cancer, find more effective biomarkers and promote early diagnosis and effective treatment. Long non-coding RNAs (lncRNAs) are generally defined as non-protein-coding RNAs with more than 200 nucleotides in length. Existing researches have shown that lncRNAs could act as sponges, guides, scaffolds, and signal molecules to influence the oncogene or tumor suppressor expressions at transcriptional, post-transcriptional, and protein levels in crucial cellular processes. Currently, the dysregulated lncRNAs are reported to involve in the pathogenesis and progression of EC. Importantly, targeting EC-related lncRNAs through genome editing, RNA interference and molecule drugs may be one of the most potential therapeutic methods for the future EC treatment. In this review, we summarized the biological functions and molecular mechanisms of lncRNAs, including oncogenic lncRNAs and tumor suppressor lncRNAs in EC. In addition, we generalized the excellent potential lncRNA candidates for diagnosis, prognosis and therapy in EC. Finally, we discussed the current challenges and opportunities of lncRNAs for EC.

## 1 Introduction

Esophageal cancer (EC), as a malignancy of the digestive tracts, is one of the most common cancers and ranks 10th in terms of morbidity and sixth in mortality across the world in 2020 cancer statistics (Sung et al., 2021). EC is typically characterized by progressive dysphagia, which tends to occur in middle-aged and elderly people especially male (Falk, 2009; Massey, 2011). There are two main histologic subtypes of EC: esophageal squamous cell carcinoma (ESCC) and esophageal adenocarcinoma (EAC). EAC is more common in western countries, while the proportion of ESCC patients is increasing in Asian countries, which also gradually occupies the main position worldwide. Meanwhile, ESCC is the second most common primary cancer in head and neck areas (Chuang et al., 2008). Compared with other HNSCC (head and neck squamous cell carcinoma) subtypes, such as oral, dental, and maxillofacial squamous cell carcinoma, ESCC has a higher incidence rate and lower survival rate (Nikbakht et al., 2020). Due to the late appearance of obvious clinical symptoms, EC is commonly diagnosed in advanced clinical stages with low five-year survival rate (less than 20%) (Dubecz et al., 2012). At present, the main therapeutic regimen for esophageal cancer is individualized and comprehensive, which is based on surgery, but the patient outcome is not satisfactory (Deng and Lin, 2018; Watanabe et al., 2020). Therefore, to further explore the occurrence and development mechanism of esophageal cancer, find more effective biomarkers, and promote early diagnosis and effective treatment, are greatly needed.

The ENCODE project showed that about 70% of the human genome is transcribed (Tay et al., 2009). However, only 1–2% RNAs encode proteins, and a majority of the human genome is transcribed into non-coding RNAs (Al-Tobasei et al., 2016). There are many types of non-coding RNAs, including microRNA (miRNA), long non-coding RNAs (lncRNAs), small nuclear RNA (snRNA), circular RNAs (circRNAs) and so on (Abraham and Meltzer, 2017; Wu and Kuo, 2020). Although most non-coding RNAs remain unstudied, there are still many ncRNAs that have been revealed to play important roles in normal cellular function and disease development (Slack and Chinnaiyan, 2019; Toden and Goel, 2022). For example, some small non-coding RNAs which are stable in blood could be used for non-invasive cancer screening (Toiyama et al., 2013; Imaoka et al., 2016). Besides, some diseases, like myotonic dystrophy, can be treated by targeting some non-coding RNAs (Wheeler et al., 2012; Levin, 2019).

LncRNAs are defined as non-coding RNAs with greater than 200 nucleotides (Cui et al., 2019). Studies showed that lncRNAs play vital roles in regulating gene expression at transcriptional, post-transcriptional and epigenetic levels in multiple cellular processes in tumors (Slack and Chinnaiyan, 2019). Interestingly, some lncRNAs have been reported to possess the small open reading frame (sORF), which could encode the small peptides to regulate multiple signaling pathways (Chen et al., 2021). Meanwhile, lncRNAs have the potential to be the biomarkers and therapeutic targets for various cancers (Dong et al., 2018). Abnormal expressions of lncRNAs were also found in the occurrence and progression of EC, and some of them were even detected in body fluid for potential biomarker (Pardini et al., 2019). Here, we summarized the biological functions of lncRNAs in the pathogenesis and progression of EC, and discussed the values of lncRNAs as biomarkers for early diagnosis, prognosis and treatment targets in EC.

## 2 Functions of lncRNAs

**FIGURE1:**
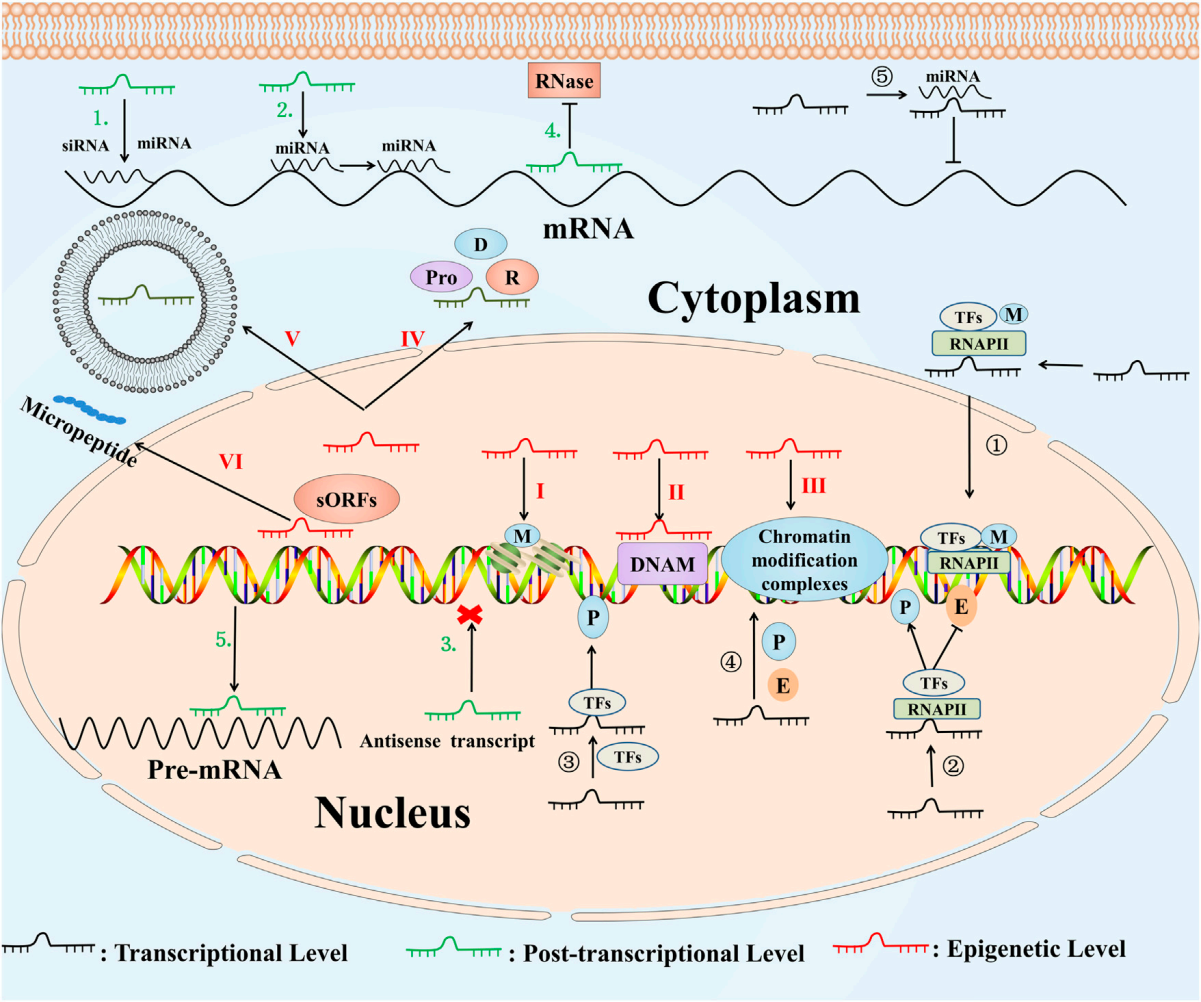
Functional molecular mechanisms of lncRNAs. ①LncRNAs change the location and modification of TFs (Hu et al., 2014). ②LncRNAs regulate the combination of the TFs, RNA polymerase Ⅱ and the promoter, enhancer (Wang and Chang, 2011). ③LncRNAs interact with DNA as a platform for TFs (Yin et al., 2015). ④LncRNAs act as competitive endogenous RNAs (Cesana et al., 2011). ⑤LncRNAs promoter may act as cis-acting enhancer elements (Liu et al., 2021b). 1) LncRNAs act as the precursor for siRNAs or miRNAs (Steck et al., 2012). 2) LncRNAs regulate the distribution of miRNA (Nachtergaele and He, 2017). 3) LncRNAs act as antisense transcripts (Liu et al., 2021a). 4) LncRNAs form the double-stranded RNA complex by binding to mRNAs (Yuan et al., 2014). 5) LncRNAs regulate the selective splicing process of pre-mRNAs (Tripathi et al., 2010). I. LncRNAs regulate histone modification through affecting the modification factors (Houseley et al., 2008). II. LncRNAs bind to DNA modification to modify the DNA methylation (Song et al., 2021). III. LncRNAs bind to chromatin modification complexes to affect chromatin structure and remodeling (Pietropaolo et al., 2021). IV. LncRNAs locate molecules by binding to DNA, RNA and proteins as a “guide” (Camier et al., 1990). V. LncRNAs participate in the formation of exosomes (Zhang et al., 2021). VI. LncRNAs that contain sORFs can encode small peptide (Wang et al., 2019c). Abbreviations: P, promoter; E, enhancer; M, modification; Pro, protein; D, DNA; R, RNA; miRNA, microRNA; TFs, transcription factors; siRNA, small-interfering RNA; RNAPII, RNA polymerase II; DNAM, DNA modification; sORFs, small open reading frames.

Growing studies showed that lncRNAs can be involved in different biological processes, for instance, epigenetic regulation, stem cell differentiation, inflammation-related diseases and tumorigenesis by mediating the transcription and translation process of protein-related coding genes (Shi et al., 2013; Hon et al., 2017; Bao et al., 2019; Zhu et al., 2019). We summarized the specific regulation mechanisms of lncRNAs in Figure 1 (Camier et al., 1990; Houseley et al., 2008; Tripathi et al., 2010; Cesana et al., 2011; Wang and Chang, 2011; Steck et al., 2012; Hu et al., 2014; Yuan et al., 2014; Yin et al., 2015; Nachtergaele and He, 2017; Wang et al., 2019c; Liu et al., 2021a; Liu et al., 2021b; Pietropaolo et al., 2021; Song et al., 2021; Zhang et al., 2021).

At transcription level, lncRNA targets the transcription factors to regulate gene transcription through trans-activation behaviors, thus affecting the gene activation, repression, cell proliferation and apoptosis (Melendez-Zajgla and Maldonado, 2021). Besides, lncRNAs could bind to DNA strand or some proteins, affecting the localization of transcription factors, decreasing the transcript elongation through inhibiting the activation of elongation factors, and inhibiting polymerase II activity via targeting the trans region (Kapranov et al., 2007).

At post-transcriptional level, lncRNAs function as the regulators of some miRNAs to regulate the gene expression, or as the competitive endogenous RNAs (ceRNAs) to regulate the expression of corresponding gene by binding miRNAs (Maass et al., 2014). For example, lncRNAs regulate the dosage compensation effect, genomic imprinting, DNA methylation, and small polypeptides coding (Sleutels et al., 2002; Ponting et al., 2009; Cai et al., 2021). Interestingly, small polypeptides encoded by small open reading frames enriched in lncRNA transcripts have been reported to play important roles in circulating extracellular vesicles (Cai et al., 2021), cancer development (Cao et al., 2021), and neuronal differentiation (Douka et al., 2021). However, the functions of most lncRNAs are still unclear and require further comprehensive study.

## 3 LncRNAs Related to Esophageal Cancer

**FIGURE 2:**
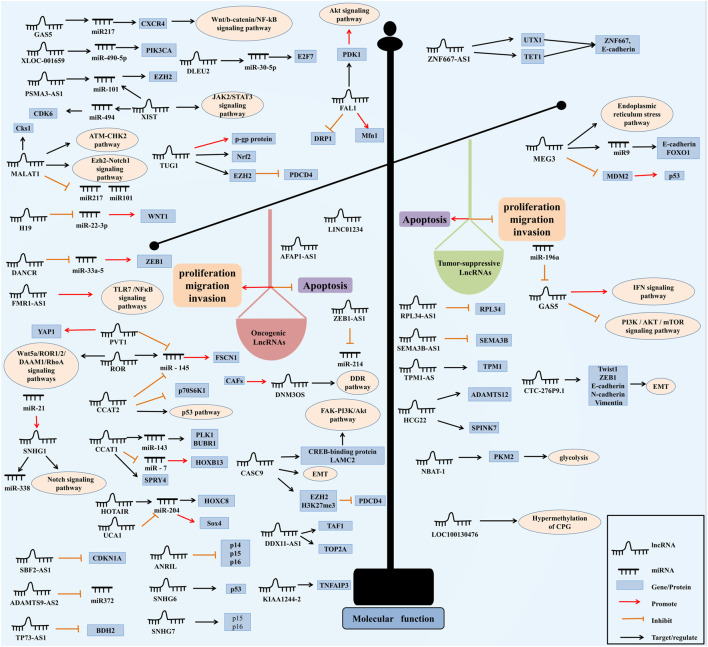
The molecular functions of lncRNAs in EC. LncRNAs function as oncogenes or tumor suppressors. On the left, lncRNAs act as oncogenes and are overexpressed in EC. On the contrary in right, lncRNAs act as tumor suppressors and are down-regulated in EC. As reported for the functional mechanisms, lncRNAs can be divided into the cis-acting lncRNAs and trans-acting lncRNAs. Cis-acting lncRNAs can regulate the chromatin state and/or expression of nearby genes: 1) the lncRNA transcript regulates the expression of nearby genes through recruiting regulatory factors (Kopp and Mendell, 2018); 2) the transcription or splicing-dependent regulation of the lncRNA execute the gene-regulation that is independent of the sequence of the RNA transcript but with structural functions (Engreitz et al., 2016); 3) Cis regulation depends on DNA elements within the lncRNA promoter (Dimitrova et al., 2014). And trans-acting lncRNAs leave the site of transcription and execute the functions: 1) lncRNAs regulate gene expression and chromatin states at genomic loci far distant from their transcription region (Rinn et al., 2007); 2) lncRNAs influence the nuclear organization and structure (Clemson et al., 2009); 3) lncRNAs interact with other proteins and/or RNA molecules (Hafner et al., 2010). In addition, lncRNA can be classified into following three categories by genic position: 1) intronic lncRNA, lncRNA within introns: mainly produced in the intron region of the coding gene (Rinn and Chang, 2012); 2) intergenic lncRNA, also known as lincRNA, mainly derived from the intergenic region of two coding genes (Ransohoff et al., 2018); 3) antisense lncRNA, mainly produced in the antisense chain of the coding gene (Rinn and Chang, 2012).

Many kinds of cancers, including EC, are associated with the imbalance of lncRNAs (Leucci et al., 2016; Hua et al., 2018; Lv et al., 2022). A study, which analyzed the lncRNAs expression profile in more than 200 EC samples from Gene Expression Omnibus (GEO) and The Cancer Genome Atlas (TCGA), demonstrated that thousands of lncRNAs were differentially expressed between EC and normal tissues, and many of them are related with patient survival time (Liu et al., 2018). LncRNAs could be divided into oncogenic lncRNAs and tumor suppressive lncRNAs based on their functions in EC, as shown in Figure 2 (St Laurent et al., 2015).

### 3.1 Oncogenic lncRNAs

As illustrated in Table 1, these lncRNAs are upregulated in EC and facilitate the malignant biological properties of EC cells. Here, we discussed some lncRNAs in more detail according to the oncogenic mechanisms of action in EC.

**TABLE 1:** EC-associated oncogenic lncRNAs.

LncRNA	Cancer Type	Up/Down	Cell Function	Underlying Mechanism	PMIDs
H19	ESCC	Up	NA	Upregulating the expression of IGF2	23,943,562
H19	ESCC	Up	Cell proliferation, metastasis, migration and invasion	Upregulating E-cadherin and downregulating vimentin and metastasis-associated protein, MMP-9	27,247,022
H19	ESCC	Up	NA	NA	31,551,175
H19	ESCC	Up	Cell proliferation, migration and stemness	Upregulating the WNT1 via down-regulating miR-22-3p expression	31,417,277
H19	EC	Up	Cell proliferation, migration, invasion, EMT and metastasis	Regulating STAT3/EZH2/β-catenin axis	31,102,664
H19	EC	Up	Cell proliferation, invasion and EMT	NA	26,171,017
FOXCUT	ESCC	Up	Cell proliferation, migration and invasion	Upregulating the expression of FOXC1	25,031,703
HNF1A-AS1	EAC	Up	Cell proliferation, migration and invasion	Regulating chromatin and nucleosome assembly and H19 induction	24,000,294
PHBP1	ESCC	Up	Cell proliferation	Upregulating expression of PHB.	28,404,970
EZR-AS1	ESCC	Up	Cell invasion	Enhancing SMYD3-dependent H3K4 methylation, thereby enhancing EZR transcription and expression	29,253,179
TTN-AS1	ESCC	Up	Cell proliferation, metastasis, apoptosis and invasion	Upregulating FSCN1 by sponging miR-133b and upregulation of mRNA-stabilizing protein HuR. And Upregulating expression of Snail1	29,101,304
HOTAIR	ESCC	Up	Cell proliferation, apoptosis, metastasis, migration and invasion	Reprogramming gene expression profile of ESCC cells, such as genes involved in cell migration and the regulation of the cell cycle	24,022,190
HOTAIR	ESCC	Up	Cell migration and invasion	Downregulating WIF-1 expression and activating Wnt/β-catenin signaling pathway	24,118,380
HOTAIR	ESCC	Up	Cell apoptosis, migration and invasion	NA	24,151,120
HOTAIR	ESCC	Up	EMT	Upregulating SNAI1 and β-catenin and downregulating E-cadherin	28,260,072
HOTAIR	EC	Up	Cell proliferation, migration, invasion and apoptosis	Binding to miR-204, regulating miR-204 and HOXC8	31,389,660
HOTAIR	EC	Up	Cell proliferation, invasion, migration and EMT	Acting as a miR-148a sponge to positively regulate Snail2 expression	28,441,714
HOTAIR	ESCC	Up	Cell invasion and EMT	Reducing the miR-130a-5p expression	31,207,321
HOTAIR	ESCC	Up	NA	NA	28,376,832
TP73-AS1	ESCC	Up	Cell proliferation	Promoting the BDH2 expression	26,799,587
CCAT1	ESCC	Up	Cell proliferation and migration	Regulating HOXB13 as a molecular decoy for miR-7, modulating the histone methylation of promoter of SPRY4	27,956,498
CCAT1	ESCC	Up	Cell proliferation and drug resistance	Regulating the miR-143/PLK1/BUBR1 signaling axis	31,544,294
CCAT2	ESCC	Up	NA	NA	25,919,911
CCAT2	EC	Up	NA	Negatively regulating the miR-145/p70S6K1 and the Akt/ERK/p70S6K1 signaling pathways	31,789,385
CCAT2	ESCC	Up	NA	NA	25,677,908
SOX2OT- s1	ESCC	Up	Cell cycle	Upregulating SOX2 and OCT4	24,105,929
SOX2OT- s2	ESCC	Up	Cell cycle	Upregulating SOX2 and OCT4	24,105,929
PlncRNA-1	ESCC	Up	Cell proliferation and apoptosis	NA	24,337,686
PSMA3-AS1	ESCC	Up	Cell proliferation, migration and invasion	Up-regulating EZH2 expression by competitively binding to miR-101	32,005,028
DLEU2	EC	Up	Cell proliferation, migration, invasion and apoptosis	Regulating miR-30e-5p/E2F7 axis	31,884,338
XLOC_001,659	ESCC	Up	Cell proliferation and invasion	Regulating miR-490-5p/PIK3CA axis	31,754,291
Linc-POU3F3	ESCC	Up	Cell proliferation and apoptosis	Downregulating POU3F3 by EZH2, recruiting DNA methyltransferases to the POU3F3 promoter; downregulating DLL1/Notch signaling	24,631,494
LincRNA-uc002yug.2	ESCC	Up	Cell apoptosis, migration and invasion	Promoting a combination of RUNX1 and alternative splicing (AS) factors in the nucleus to produce more RUNX1a, the short isoform and inhibitor of RUNX1. And reducing CEBPα expression	25,486,427
PEG10	ESCC	Up	Cell proliferation, invasion and apoptosis	Downregulating expression of PEG10	25,591,808
ANRIL	ESCC	Up	Cell proliferation	Reducing the expression of p15 and TGFβ1	24,747,824
XIST	EC	Up	Cell proliferation, migration, invasion, and apoptosis	Regulating miR-494/CDK6 axis through JAK2/STAT3 signal pathway	30,551,480
XIST	ESCC	Up	Cell proliferation, migration and invasion	Regulating miR-101/EZH2 axis	29,100,288
FAL1	ESCC	Up	Cell apoptosis	By the mitochondrial pathway	30,501,006
FAL1	EC	Up	Cell proliferation, invasion and apoptosis	Activating AKT pathway via targeting PDK1	30,178,844
UCA1	ESCC	Up	NA	NA	30,002,691
UCA1	EC	Up	Cell proliferation	Regulating Sox4 expression by competitively binding miR-204	27,667,646
UCA1	EC	Up	NA	NA	31,414,398
UCA1	EC	Up	Cell proliferation, invasion, migration and EMT	Inhibiting miR-498 expression and thereby increasing ZEB2 expression	31,387,451
UCA1	ESCC	Up	Cell proliferation, migration and invasion	NA	25,550,835
SPRY4-IT1	ESCC	Up	Cell proliferation, migration and invasion	NA	24,810,925
SPRY4-IT1	ESCC	Up	Cell proliferation, migration and invasion	NA	26,883,252
SPRY4-IT1	ESCC	Up	EMT	Increasing expression of vimentin and fibronectin with a concomitant decrease of E-cadherin and ZO-1. And increasing transcription, expression, and nuclear localization of Snail and TFG-β signaling pathway	27,250,657
SPRY4-IT1	ESCC	Up	Cell proliferation	Upregulating expression of ZNF703	27,453,415
TUG1	ESCC	Up	Cell proliferation and migration	NA	25,366,138
TUG1	ESCC	Up	Cell proliferation, apoptosis, migration, and invasion	Downregulating miR-498 and upregulating XBP1	32,305,055
TUG1	ESCC	Up	Cell proliferation, apoptosis and invasion	Regulating PLK1 expression by sponging miR-1294	33,009,634
TUG1	ESCC	Up	Cell proliferation, migration, invasion and apoptosis	Regulating miR-148a-3p/MCL-1/Wnt/β-catenin axis	31,742,924
GAS5	EC	Up	Cell viability, migration, invasion and apoptosis	Regulating miR-301a	29,386,089
TUG1	ESCC	Up	Cell proliferation and invasion	Downregulating miR-498 and upregulating CDC42	32,139,664
MALAT1	ESCC	Up	Invasion, migration and EMT	Regulating Ezh2-Notch1 signaling pathway	29,916,899
MALAT1	EC	Up	Cell proliferation, migration and invasion	NA	27,470,544
MALAT1	ESCC	Up	Cell proliferation, migration and invasion	Upregulating p21 and p27 expression and downregulating B-MYB expression	25,538,231
MALAT1	ESCC	Up	Cell proliferation, apoptosis, migration and invasion	Inactivating ATM-CHK2 pathway	25,613,496
MALAT1	ESCC	Up	Cell proliferation, apoptosis, migration and invasion	NA	26,493,997
MALAT1	ESCC	Up	Cell proliferation, apoptosis and migration	Upregulating expression of β-catenin, Lin28 and Ezh2 genes	27,015,363
MALAT1	ESCC	Up	Cell migration, stemness and chemoresistance	Enhancing YAP protein expression and increasing YAP transcriptional activity	31,116,509
MALAT1	EC	Up	Cell viability, migration, EMT and invasion	Downregulating miR-1-3p and activating CORO1C/TPM3 signaling	32,468,237
ESCCAL-1	ESCC	Up	Cell apoptosis and invasion	NA	25,885,227
DANCR	ESCC	Up	Cell proliferation and migration	Regulating miR-33a-5p	31,401,160
DANCR	ESCC	Up	Cell proliferation, migration, invasion and apoptosis	NA	29,997,918
TINCR	ESCC	Up	Cell proliferation, apoptosis, migration and invasion	NA	26,833,746
POU6F2-AS2	ESCC	Up	Cell apoptosis	Reducing DNA damage and promoting cells survival after ionizing radiation	27,033,944
FMR1-AS1	ESCC	Up	Cell proliferation, migration and apoptosis	Promoting c-Myc expression through interacting with TLR7 and activating NF-κB signaling	30,736,860
SBF2-AS1	ESCC	Up	Cell proliferation, migration and invasion	Decreasing the CDKN1A expression	29,552,140
CASC9	ESCC	Up	Migration, invasion and metastasis	Activating the FAK-PI3K/Akt signaling pathways through LAMC2	29,511,340
CASC9	ESCC	Up	Cell proliferation, migration, invasion and EMT	NA	29,424,900
CASC9	ESCC	Up	Cell proliferation and apoptosis	Regulating PDCD4 expression via EZH2	28,854,977
CASC9	ESCC	Up	Cell migration and invasion	NA	27,431,358
LincRNA-NR_024,015	ESCC	Up	Cell proliferation and invasion	Inhibiting expression of miR-526b	27,583,835
HOTTIP	ESCC	Up	Cell proliferation, apoptosis, migration, invasion and EMT	NA	27,806,322
HOTTIP	ESCC	Up	Cell proliferation, metastasis, EMT and invasion	Downregulating miR-30b, thereby upregulating SNAIL1 and HOXA13. And directly bounding WDR5 and driving histone H3 lysine 4 trimethylation and HOXA13 gene transcription	28,534,516
ATB	ESCC	Up	Cell proliferation and migration	Downregulating miR-200b and upregulating Kindlin-2	28,640,252
Linc00460	ESCC	Up	Cell proliferation and apoptosis	CBP/P300 binding to linc00460 promoter activates linc00460 transcription through histone acetylation	28,939,763
GHET1	ESCC	Up	Cell proliferation, apoptosis, migration and invasion	Upregulating expression of vimentin and N-cadherin while downregulating expression of E-cadherin	28,983,895
PVT1	EAC	Up	Cell proliferation and invasion	PVT1 and YAP1 are associated and positively regulate each other	31,601,234
PVT1	EC	Up	Cell viability, migration, invasion and apoptosis	Inhibiting miR-145 expression by upregulating FSCN1	31,369,196
PVT1	ESCC	Up	Cell proliferation and migration	Regulating miR-203/LASP1 axis	28,404,954
LUCAT1	ESCC	Up	Cell proliferation, apoptosis, migration and invasion	Inhibiting DNMT1 ubiquitination through UHRF1 and inhibiting expression of tumor suppressors through DNA methylation	29,247,823
NEAT1	ESCC	Up	Cell viability and invasion	Downregulating miR-129 and upregulating CTBP2	29,147,064
ROR	ESCC	Up	Cell proliferation and chemoresistance	Downregulating miR-15b, miR-33a, miR-129, miR-145, and miR-206 and upregulating SOX9	29,237,490
ROR	ESCC	Up	Cell proliferation and apoptosis	Downregulating miR-204-5p and upregulating MDM2; enhancing the ubiquitination level of p53	31,541,467
ROR	ESCC	Up	Migration and invasion	Regulating miR-145/FSCN1 axis	29,430,188
PCAT1	ESCC	Up	Cell proliferation	Binding to and sponging miR-326, a tumor suppressor	31,273,188
PCAT1	EC	Up	Cell proliferation and chemoresistance	NA	29,314,203
LINC01503	ESCC	Up	Cell proliferation, migration and invasion	Inhibiting ERK2 dephosphorylation by DUSP6, leading to activation of ERK signaling via MAPK. And disrupting interaction between EBP1 and the p85 subunit of PI3K, increasing AKT signaling	29,454,790
Linc-UBC1	ESCC	Up	Cell migration and invasion	Upregulating EZH2 and downregulating E-cadherin	29,552,776
MIR31HG	ESCC	Up	Cell proliferation, migration and invasion	Upregulating expression of Furin and MMP1	29,605,445
MIR31HG	ESCC	Up	Cell proliferation and apoptosis	Downregulating miR-34a and upregulating c-Met.	32,502,839
NMR	ESCC	Up	Cell apoptosis, migration and invasion	Upregulating MMP3 and MMP10 expression through ERK1/2 activation	29,763,634
DUXAP8	ESCC	Up	Cell proliferation and invasion	Activating Wnt/β-catenin pathway	29,771,416
SNHG16	ESCC	Up	Cell proliferation, apoptosis and invasion	Activating Wnt/β-catenin pathway	29,949,155
LINC01296	ESCC	Up	Cell proliferation, migration and invasion	NA	30,058,683
FTH1P3	ESCC	Up	Cell proliferation, migration and invasion	Downregulating expression of Sp1 and NF-kB (p65)	30,119,232
LINC01617	ESCC	Up	Cell proliferation, migration and invasion	Activating Akt pathway	30,120,975
LINC00657	ESCC	Up	Cell proliferation and migration	Downregulating miR-615-3p and upregulating JunB	30,227,324
DLX6-AS1	ESCC	Up	Cell proliferation, apoptosis, migration, invasion and EMT	NA	30,592,268
LINC00152	ESCC	Up	Cell proliferation and apoptosis	Downregulating miR-153-3p and upregulating FYN.	30,784,933
LBX2-AS1	ESCC	Up	Cell migration and EMT	Enhancing the stability of ZEB1 and ZEB2	30,824,187
LINC01980	ESCC	Up	Cell proliferation and apoptosis	Upregulating the expression of GADD45A	30,935,686
LINC01980	ESCC	Up	Cell proliferation, migration, invasion and EMT	Downregulating miR-190a-5p and upregulating MYO5A	32,325,088
ATB	ESCC	Up	Cell proliferation and invasion	Upregulating the expression of IL-11	30,954,889
LINC00473	ESCC	Up	Radioresistance	Downregulating miR-374a-5p and upregulating SPIN1	31,017,716
LINC00857	EAC	Up	Cell proliferation, apoptosis, migration and invasion	Upregulating the level of MET, STAT3, c-Myc and p-CREB proteins	31,085,800
Erbb4-IR	ESCC	Up	Cell proliferation and apoptosis	Downregulating miR-145	31,119,810
MNX1-AS1	ESCC	Up	Cell proliferation, apoptosis, migration and invasion	Downregulating miR-34a and upregulating SIRT1	31,170,665
LINC00184	ESCC	Up	Cell proliferation, migration and invasion	Enhancing the promoter methylation of PTEN and the Akt phosphorylation	31,201,145
MIR22HG	EAC	Up	Cell proliferation, apoptosis, invasion and migration	Increasing the expression of STAT3/c-Myc/p-FAK proteins	31,291,201
LSINCT5	ESCC	Up	Cell proliferation, invasion and migration	NA	31,298,370
HAGLR	ESCC	Up	Cell proliferation, invasion, EMT and migration	Downregulating miR-143-5p and upregulating LAMP3	31,311,326
PANDA	ESCC	Up	Cell proliferation and apoptosis	Increasing E2F1, cyclinD1, cyclinD2, cyclinE1 and Bcl-2 expression; drifting away from NF-YA to promote expression of NF-YA-E2F1	31,495,606
NR2F1-AS1	ESCC	Up	Cell proliferation, invasion, EMT and migration	Activating Hedgehog signaling pathway a by upregulating GLI2 to upregulate NR2F1 expression	31,530,388
PTCSC1	ESCC	Up	Cell proliferation and migration	Activating Akt pathway	32,971,114
LOC440173	ESCC	Up	Cell proliferation, invasion, EMT and migration	Downregulating miR-30d-5p and upregulating HDAC9	33,079,409
LINC00491	ESCC	Up	Cell proliferation, invasion and apoptosis	NA	33,537,830
EIF3J-AS1	ESCC	Up	Cell proliferation and invasion	Downregulating miR-373-3p and upregulating AKT1	32,811,869
LINC00634	ESCC	Up	Cell viability and apoptosis	Downregulating miR-342-3p and upregulating Bcl2L1	32,583,748
LOC100133669	ESCC	Up	Cell proliferation	Binding to Tim50 and upregulating protein level of Tim50 through inhibiting ubiquitination	32,130,753
LINC01234	EC	Up	Cell proliferation and apoptosis	Downregulating miR-193a-5p and upregulating CCNE1	32,130,660
LINC01234	EC	Up	Cell proliferation, migration, invasion and apoptosis	NA	30,519,325
SNHG1	ESCC	Up	NA	Regulating miRNA-21	32,021,418
SNHG1	ESCC	Up	Cell proliferation, invasion and EMT	Activating the Notch signaling pathway	29,081,407
SNHG1	EC	Up	Cell proliferation and apoptosis	Regulating miR-338	28,423,738
SNHG6	ESCC	Up	Cell proliferation, migration and invasion	NA	30,899,408
SNHG6	ESCC	Up	Cell proliferation and apoptosis	NA	29,616,119
SNHG6	ESCC	Up	Cell proliferation, invasion and migration	Downregulating miR-186-5p and upregulating HIF1α	31,853,782
SNHG7	EC	Up	Cell proliferation and apoptosis	NA	29,771,415
AFAP1-AS1	ESCC	Up	Cell proliferation and apoptosis	NA	27,577,754
CASC11	ESCC	Up	Cell proliferation and apoptosis	Downregulating the expression of KLF6	31,696,474
LINC00473	ESCC	Up	Cell proliferation, invasion and EMT	Downregulating miR-497-5p and upregulating PRKAA1	31,584,290
ZEB1-AS1	ESCC	Up	Cell proliferation and invasion	Downregulating the expression of ZEB1	31,638,344
EGFR-AS1	ESCC	Up	Cell invasion and migration	Downregulating miR-145 and upregulating ROCK1	31,702,393
HERES	ESCC	Up	Cell proliferation, invasion and migration	Regulating the expression of CACNA2D3, SFRP2, and CXXC4 to activate Wnt signaling pathways through interacting with EZH2	31,732,666
ZFAS1	ESCC	Up	Cell proliferation, apoptosis, invasion and migration	Downregulating miR-124 and upregulating STAT3	31,775,815
LINC01518	ESCC	Up	Cell proliferation and apoptosis	Downregulating miR-1-3p and upregulating PIK3CA to activate AKT pathway	31,810,385
LINC00963	ESCC	Up	Cell proliferation and invasion	Downregulating miR-214-5p and upregulating RAB14	31,957,829
VPS9D1-AS1	ESCC	Up	Cell proliferation, invasion and migration	Regulating the expression of β-catenin and c-Myc	34,659,577
DDX11-AS1	ESCC	Up	Cell proliferation, invasion, migration and EMT	Sponging miR-30d-5p, thus upregulating the expression of SNAI1 and enhancer of ZEB2	34,866,524
HCP5	ESCC	Up	Cell proliferation, apoptosis, invasion and migration	Regulating the miR-139-5p/PDE4A axis and activating the PI3K-AKT- mTOR signaling pathway	34,190,001
KCNQ1	ESCC	Up	Cell proliferation, invasion and migration	Activating the PI3K/AKT pathway via inhibiting the repression of miR-133b and indirectly upregulating EGFR.	33,909,822
FAM225A	ESCC	Up	Cell proliferation, invasion and migration	Sponging miR-197-5p, thus upregulating the NONO expression and activating the TGF-β pathway	33,442,405
NCK1-AS1	ESCC	Up	Cell proliferation, invasion and migration	Upregulating the expression of TGF-β1	35,311,444
LINC01535	ESCC	Up	Cell proliferation, invasion and migration	Activating the JAK/STAT3 pathway	32,329,845

EC, esophageal cancer; ESCC, esophageal squamous cell carcinoma; NA, Not Available.

#### 3.1.1 Oncogenic lncRNAs Involving Wnt Signaling Pathway

Wnt signaling is one of the crucial pathways regulating cell biology (Clevers and Nusse, 2012). The dysregulation of Wnt signaling has been described in various cancers, including lung cancer (Stewart, 2014), colorectal cancer (Zhan et al., 2017), etc. Currently, several studies revealed that lncRNAs can affect the malignant behaviors of EC through Wnt signaling pathway.

Small nucleolar RNA host gene 16 (SNHG16), Colon cancer-associated transcript 2 (CCAT2) and FEZ family zinc finger 1 antisense RNA 1 (FEZF1-AS1) were upregulated in EC cells and the knockdown of them inhibited the malignant behaviors of EC *via* the reduction of β-catenin expression in Wnt signaling pathway (Cai et al., 2015; Xu et al., 2018b; Han et al., 2018; Yang et al., 2019a; Wang and Wang, 2019). Ma et al. (2021) reported that lncRNA VPS9D1 antisense RNA1 (VPS9D1-AS1) was overexpressed in ESCC cell lines and tissues compared with normal controls. Silencing of VPS9D1-AS1 could inhibit the proliferation, invasion, and migration ability *in vitro*. Mechanically, VPS9D1-AS1 knockdown can inhibit the Wnt/β-catenin pathways through regulating the expression of β-catenin and c-Myc (Ma et al., 2021). Guo et al. (2021) revealed that DDX11 antisense RNA 1 (DDX11-AS1) overexpression enhanced cell proliferation, invasion, migration, and epithelial - mesenchymal transition (EMT) process of EC through activating the Wnt/β-catenin pathway *via* targeting miR-30d-5p. Meanwhile, DDX11-AS1 could act as a ceRNA through sponging miR-30d-5p, thus upregulating the expression of SNAI1 and Zinc Finger E-Box Binding Homeobox 2 (ZEB2) (Guo et al., 2021). You et al. (2019) found that LncRNA HERES, as an oncogene, can activate Wnt signaling by interacting with enhancer of zeste homolog 2 (EZH2) *via* its G-quadruple structure-like motif. Additionally, TUG1 exerts oncogenic ability though activating the Wnt/β-catenin pathway *via* miR-148a-3p/myeloid cell leukemia-1 (MCL-1) axis (Tang et al., 2020). Similarly, lncRNA growth arrest-specific transcript 5 (GAS5), as a molecular sponge, can regulate miR-301a, thus enhancing C-X-C chemokine receptor 4 (CXCR4) expression and activating NF-κB pathways and the Wnt/β-catenin pathway in EC (Li et al., 2018). Yang et al. (2021) found that knockdown of CCAT2 inhibited the mRNA and protein levels of wnt-induced-secreted-protein-1 (WISP1), β-catenin, and the mRNA levels of their downstream target genes in ESCC. Similarly, HOX transcript antisense intergenic RNA (HOTAIR) directly inhibited the Wnt inhibitory factor 1 (WIF-1) expression by promoting the histone methylation on H3K27 in the promoter region, thus activating the Wnt/β-catenin signaling pathway in ESCC (Ge et al., 2013).

Given the above, many lncRNAs play an important role in the occurrence and development of EC by regulating the Wnt signaling pathway.

#### 3.1.2 Oncogenic lncRNAs Involving PI3K/AKT Signaling Pathway

PI3K/AKT signaling pathway has been reported to be frequently activated in a variety of tumors and has been considered as a potential therapeutic target (Aoki and Fujishita, 2017). Here, we showed that some lncRNAs can regulate the EC progression through PI3K/AKT signaling pathway.

LINC00152 was up-regulated in EC tissues, and knockdown of LINC00152 significantly inhibited the cell proliferation of EC by decreasing the expression levels of PI3K and AKT (Ding et al., 2020). Another study showed that LncRNA HLA complex P5 (HCP5) promoted malignant behaviors of ESCC through regulating the miR-139-5p/phosphodiesterase 4A (PDE4A) axis and activating the PI3K-AKT-mammalian target of rapamycin (mTOR) signaling pathway (Xu et al., 2021b). Similarly, overexpression of LINC01518, PTCSC1, LINC01617, and methyltransferase like 3 (METTL3) can also enhance the malignant behaviors of EC cells through activating the AKT pathway (Zhang et al., 2018a; Zhang et al., 2019b; Liu et al., 2020c; Hou et al., 2020). Liang et al. (2018) found that focally amplified lncRNA (FAL1) promoted the proliferation of EC cells by stimulating the AKT pathway *via* regulating the 3-phosphoinositide-dependent kinase 1 (PDK1). Importantly, EIF3J-AS1, as an oncogene, can sponge the miR-373-3p to up-regulate mRNA level of AKT1 in EC (Wei et al., 2020). Another study showed that ESCC-related lncRNAs transcript 1 (ESSCAL-1) downregulation can induce the cell apoptosis by the downregulation of the PI3K/AKT signaling *via* miR-590-3p/apolipoprotein mRNA-editing enzyme catalytic polypeptide 3 protein G (APOBEC3G) axis (Liu et al., 2020a). Xu et al. (2021a) reported that KCNQ1 overlapping transcript 1 (KCNQ1OT1) can activate the PI3K/AKT pathway *via* inhibiting the repression of miR-133b and indirectly upregulating epidermal growth factor receptor (EGFR). In addition, LINC00184 regulated the glycolysis and mitochondrial oxidative phosphorylation (OXPHOS) of EC cells through inducing the phosphorylation of Akt (Li et al., 2019).

In conclusion, numerous lncRNA dysregulation participates in the occurrence and development of EC through affecting the PI3K/AKT pathway.

#### 3.1.3 Oncogenic lncRNAs Involving TGF-β Signaling Pathway

TGF-β signaling pathway has been showed to regulate numerous cellular functions, including cell proliferation, apoptosis, invasion, and migration (Colak and Ten Dijke, 2017). Current studies revealed that some lncRNAs can regulate the EC progression by affecting the TGF-β signaling pathway.

Zhu et al. (2021) found that lncRNA FAM225A facilitated the ESCC development through sponging miR-197-5p, thus upregulating the non-POU domain-containing octamer-binding protein (NONO) expression and activating the transforming growth factor-β (TGF-β) pathway in ESCC cells. Another study revealed that NCK1-AS1 may promote ESCC progression by upregulating the expression of TGF-β1 (Fu et al., 2022). Similarly, ANRIL knockdown inhibit EC cell proliferation by increasing the expression of p15 *via* TGFβ1 (Chen et al., 2014).

In brief, some lncRNAs can regulate the TGF-βpathway to affect the EC development.

#### 3.1.4 Oncogenic lncRNAs Involving Other Regulatory Mechanisms

We also discussed lncRNAs promote the tumorigenesis and development of EC through other regulatory mechanisms. Here, we mainly discussed the HOX transcript antisense intergenic RNA (HOTAIR), MALAT1 LINC01535 and BRAF-activated non-coding RNA (BANCR), which have got wide attention in multiple studies.

Several studies have revealed that HOTAIR, as a competing endogenous RNA, plays the oncogenic role through the lncRNA-miRNA-mRNA function network in EC. Ren et al. (2016) reported that HOTAIR was abnormally increased in ESCC cells and could facilitate ESCC occurrence through sponging miR-1 and upregulating CCND1. Xu et al. found that HOTAIR may promote epithelial–mesenchymal transition (EMT) through acting as the miR-148a sponge to facilitate the expression of snail2 (Xu and Zhang, 2017). It was reported that the up-regulation of homeobox C8 (HOXC8) was observed in a variety of cancer types and involved in tumor formation (Li et al., 2020a; Jiang et al., 2021). Importantly, Wang et al. (2019a) showed that HOTAIR could regulate HOXC8 by specifically binding to miR-204 as a competing endogenous RNA. CC motif chemokine ligand 18 (CCL18) has crucial roles in tumor progression and metastasis (Cardoso et al., 2021). Wang et al. (2019e) revealed that HOTAIR upregulated by CCL18 advanced malignant progression of ESCC through miR-130a-5p-zinc finger E-box binding homeobox 1 (ZEB1) axis. Hexokinase 2 (HK2) expression is increased in tumors and contributes to aerobic glycolysis. Ma et al. (2017) found that HOTAIR promoted HK2 expression by sponging miR-125/miR-143, thus facilitating the tumorigenesis of ESCC.

Researchers have also revealed that MALAT1 played a crucial role in the tumorigenesis and development of EC. MALAT1 was significantly up-regulated in EC tissues and cells than the normal controls (Huang et al., 2016). A study showed that the up-regulation of MALAT1 in advanced stage ESCC tissues may facilitate ESCC growth by the ataxia-telangiectasia mutated (ATM)/checkpoint kinase 2 (CHK2) pathway dephosphorylation (Hu et al., 2015). MALAT1 knockdown suppressed the invasion, migration and EMT of EC. Mechanically, MALAT1 could target and sponge the miR-1-3p, thus affecting the coronin-1C (CORO1C)/tropomyosin3 (TPM3) pathway in EC (Li et al., 2020c). Besides, knockdown of MALAT1 inhibited the proliferation, migration and tumor sphere formation of tumors cells by decreasing the expression of β-catenin, Lin28 and EZH2 in ESCC (Wang et al., 2016a). Another study found that MALAT1 could promote the EMT and metastasis of EC cells through Ezh2-Notch1 signaling pathway (Chen et al., 2018). Importantly, down-regulation of MALAT1 by miR-101 and miR-217 through a post-transcriptional regulation mechanism inhibited proliferation, invasion, and migration of ESCC cells (Wang et al., 2015). Subsequently, Wang et al. (2021b) found that N 6-methyladenosine (m^6^A) modification of MALAT1 promotes the metastasis ability of ESCC through reshaping nuclear speckles (NSs). The transcription events in downstream activated by NSs is partially intermediated through the binding of YTH-domain-containing protein 1 (YTHDC1) onto MALAT1-Δm^6^A. Interestingly, artificially tethering YTHDC1 onto MALAT1-Δm^6^A ESCC cells leads to the restoring of migration ability (Wang et al., 2021b), indicating that post-transcriptional modification may affect the oncogenic activity of MALAT1 in EC.

LINC01535 promoted the proliferation and decreased the apoptosis in ESCC cells *via* activating the JAK/STAT3 pathway (Fang et al., 2020). Another study showed that small nucleolar RNA host gene 20 (SNHG20) played the oncogenic role through enhancing the growth and metastasis of ESCC through the ataxia telangiectasia-mutated kinase (ATM)-JAK-programmed cell death 1 ligand 1 (PD-L1) axis (Zhang et al., 2019a).

BRAF-activated non-coding RNA (BANCR) was revealed to enhance the cell proliferation, invasion and migration of ESCC by Raf/MEK/ERK signaling (Yu et al., 2021). Song et al. (2020) also found that BANCR can promote the malignant behaviors of ESCC and BANCR downregulation inhibited the ESCC development by inactivating the insulin like growth factor 1 receptor (IGF1R)/Raf/MEK/ERK axis by sponging the miR-338-3p.

To sum up, the oncogenic effects of lncRNAs in EC are multi-channel through various cell pathways.

### 3.2 Tumor-Suppressive lncRNAs

Deregulation of tumor suppressors plays important roles during carcinogenesis and tumor progression. In-depth understanding of tumor suppressors can serve new ideas for tumor-targeted therapy. It can promote EC occurrence and development once the tumor suppressive lncRNAs are downregulated. We summarize the information about tumor suppressive lncRNAs in Table 2. In this section, we discussed the tumor-suppressive role of some lncRNAs according to the mechanisms of action in EC.

**TABLE 2:** EC-associated tumor suppressive lncRNAs.

LncRNA name	Cancer Type	Up/Down	Cell Function	Underlying Mechanism	PMIDs
91H	ESCC	Down	NA	Downregulating IGF2	24,706,416
Epist	ESCC	Down	Cell migration and invasion	Upregulating PITX1 expression and downregulating TERT expression	26,158,411
LET	ESCC	Down	Cell proliferation, apoptosis, migration and invasion	Activating p53 protein	26,935,396
LET	ESCC	Down	Cell proliferation and migration	MiR-548k targets and represses expression of lncRNA-LET and down-regulates p53 and up-regulates NF90	29,126,868
UCA1	ESCC	Down	Cell proliferation, apoptosis, migration and invasion	Inhibiting Wnt signaling pathway. Upregulating DKK1 and downregulating C-myc. And reducing β-catenin levels in both total and nuclear proteins	27,267,823
RP11-766N7.4	ESCC	Down	Cell migration, invasion and EMT	NA	28,157,654
NKILA	ESCC	Down	Cell proliferation and migration	Inhibiting phosphorylation of IκBα, suppressing p65 nuclear translocation and downregulating expression of NF-κB target genes	29,348,395
NKILA	ESCC	Down	Cell migration and invasion	Inhibiting MMP14 expression by inhibiting IκBα phosphorylation and NF-κB activation	29,379,981
TUSC7	ESCC	Down	Cell proliferation, apoptosis and chemoresistance	Downregulating miR-224 and increasing DESC1 expression	29,530,057
FER1L4	ESCC	Down	Cell proliferation, apoptosis and invasion	NA	29,771,417
LINC00261	ESCC	Down	Cell proliferation, apoptosis and chemoresistance	Increase methylation of DPYD promoter through recruitment of DNMT. And decreasing DPYD activity	30,226,808
NEF	ESCC	Down	Cell proliferation, migration and invasion	Downregulating expression levels of Wnt/β-catenin pathway-related proteins	30,402,846
HAND2-AS1	ESCC	Down	Cell proliferation, migration and invasion	Downregulating miR-21	30,520,131
LINC00675	ESCC	Down	Cell proliferation, apoptosis, migration, invasion and EMT	Inhibiting Wnt/β-catenin pathway	30,556,869
MEG3	ESCC	Down	Cell proliferation, migration and invasion	Downregulating miR-4261, upregulating DKK2 and blocking the Wnt/β-catenin signaling pathway	30,990,378
IRF1-AS	ESCC	NA	Cell proliferation and apoptosis	Activating IRF1 transcription through interacting with ILF3 and DHX9. And IRF1 binds to the IRF1-AS promoter and activates IRF1-AS transcription	31,173,852
PGM5-AS1	ESCC	Down	Cell proliferation, migration and invasion	PGM5-AS1 was transcriptionally activated by p53 and it could directly interact with and downregulate miR-466 to elevate PTEN expression	31,185,143
ADAMTS9-AS2	ESCC	Down	Cell proliferation, invasion and migration	Enhancing the methylation of CDH3 promoter via DNMT1/DNMT3	31,621,118
ZNF667-AS1	ESCC	Down	Cell viability, migration and invasion	Increasing the expression of ZNF667; recruiting TET1 and interacting UTX to decrease histone H3K27 tri-methylation to activate the expression of ZNF667 and E-cadherin	31,804,468
NBAT-1	ESCC	Down	Cell proliferation	Downregulating the expression of PKM2	31,632,565
RPL34-AS1	ESCC	Down	Cell proliferation, invasion and migration	Downregulating the expression of RPL34	31,574,377
SEMA3B-AS1	ESCC	Down	Cell viability and invasion	Upregulating the protein expression of SEMA3B	30,915,595
GAS5	ESCC	Down	Cell proliferation and migration	Decreasing the expression of PI3K and phosphorylation levels of Akt and mTOR.	30,368,517
GAS5	ESCC	Up	Cell proliferation, invasion and migration	Induced by IFN responses via JAK-STAT pathway; activating the IFN responses	29,745,062
GAS5	ESCC	Down	Cell proliferation	Decreased by miR-196a and binding to Ago2	29,170,131
CTC-276P9.1	ESCC	Down	Cell proliferation and invasion	NA	29,524,086
TPM1-AS	ESCC	Down	Cell migration	Interacting with RBM4 and hindering the binding of RBM4 to TPM1 pre-mRNA and inhibiting RBM4 to promote endogenous exon 2a inclusion of TPM1	28,754,317
MEG3	ESCC	Down	Cell proliferation and invasion	Downregulating miR-9 and increasing E-cadherin and FOXO1 expression	28,539,329
MEG3	ESCC	Down	Cell proliferation, apoptosis and metastasis	Activating p53 and its target genes by downregulating MDM2	27,778,235
MEG3	ESCC	Down	Cell proliferation and apoptosis	Increasing the expression of ER stress-related proteins (GRP78, IRE1, PERK, ATF6, CHOP and cleaved-caspase-3)	28,405,686
LOC100130476	ESCC	Down	Cell proliferation and invasion	NA	27,338,851

ESCC, esophageal squamous cell carcinoma; NA, Not Available.

#### 3.2.1 Tumor-Suppressive lncRNAs Involving Wnt Signaling Pathway

Overexpression of lncRNA-neighboring enhancer of FOXA2 (NEF), growth-arrest associated lncRNA 1 (GASL1), LINC00675 inhibited cell proliferation, invasion and migration through decreasing the expression of β-catenin in ESCC (Zhang et al., 2018b; Zhong et al., 2018; Ren et al., 2021). Urothelial carcinoma-associated (UCA1) expression was significantly lower in ESCC tissues than adjacent normal tissues. And mRNA microarray analysis indicated the overexpression of UCA1 can inhibit the Wnt signaling pathway through reducing the β-catenin (active form) levels (Wang et al., 2016b). Another study displayed that miR-4261, which could promote ESCC cell function *in vitro*, is one of maternally expressed gene 3 (MEG3) targets. The anti-tumor effect of MEG3 in ESCC was related to MEG3-miR-4261 axis regulating the dickkopf-2 (DKK2) and Wnt/β-catenin signaling (Ma et al., 2019).

#### 3.2.2 Tumor-Suppressive lncRNAs Involving Other Regulatory Mechanisms

Here, we discussed other regulatory mechanisms lncRNAs, which can inhibit the tumorigenesis and development of EC. We mainly discussed the regulatory mechanisms regulated by MEG3 and GAS5 in EC.

The results of Li’s study proposed that MEG3 may inhibit EMT and EC cell function through downregulating phosphoserine aminotransferase 1 (PSAT1) and restraining the PSAT1 dependent GSK-3β/Snail signaling pathway (Li et al., 2020b). In addition, MEG3 was found to be significantly reduced in ESCC tissues, which was mediated by DNA methylation. And ectopic expression of MEG3 could promote ESCC cell apoptosis and inhibit cell proliferation and metastasis. Tumor protein 53 (TP53) is one of best-known tumor suppressors. One way of MEG3 inhibiting EC is by inducing p53 activation due to mouse double minute 2 (MDM2) downregulation (Lv et al., 2016). Moreover, lower expression of MEG3 predicted shorter survival by analyzing EC dataset from TCGA (Lv et al., 2016). Huang et al. (2017) also discovered MEG3 expression was diminished in ESCC tissue. MEG3 could suppress EC cell growth and induce apoptosis through activating endoplasmic reticulum stress pathway because the overexpression of MEG3 increased endoplasmic reticulum stress - related proteins (ATF6, GRP78, CHOP, PERK, IRE1, and cleaved caspase-3) expression (Huang et al., 2017). Dong et al. (2017) observed that there were hypermethylation of MEG3 both in ESCC tissue and EC cell lines, and it is crucial to MEG3 gene silencing. They also found that MEG3 may regulate forkhead box other 1 (FOXO1) and E-cadherin expression through competitively binding miR-9 (Dong et al., 2017).

In Wang’s study, GAS5 was lower in tumor tissues than that in healthy tissues, and the serum GAS5 level in EC patients was significantly lower than that in normal controls. Overexpression of GAS5 inhibited EC cell proliferation and migration by inactivating PI3K/AKT/mTOR signal pathway (Wang et al., 2018). Huang et al. (2018) found that tumor-suppressive lncRNA GAS5 was regulated by interferon (IFN) responses through the JAK/STAT signaling pathway. A study discovered the level of NBAT-1 was lower in EC tissue than adjacent normal tissues. And NBAT-1 overexpression could inhibit EC cell proliferation and tumor glycolysis partially by reducing pyruvate kinase M2 (PKM2) protein expression (Zhao et al., 2019). According to Ke et al. (2018)’s study, they also found that the expression of GAS5 was downregulated in ESCC tissue and ESCC cell. GAS5 overexpression may suppress ESCC cell function through inducing cell cycle arrest at G2/M stage by activating the ATM-CHK2 pathway and affecting EMT-associated proteins (Ke et al., 2018).

## 4 LncRNAs in Diagnosis, Prognosis and Therapy of Esophageal Cancer

LncRNAs play crucial roles in both cellular physiological and pathological processes, including in the pathogenesis of various tumors and often reveal a higher cell/tissue specificity than mRNA (Derrien et al., 2012; De Falco et al., 2021; Turai et al., 2021), so they were suggested as potential valuable diagnostic, prognostic factors even therapeutic targets. Below, we summarize the excellent potential lncRNA candidates for diagnosis, prognosis and therapy in EC.

### 4.1 LncRNAs in Esophageal Cancer Diagnosis

The treatment effect is not satisfactory for EC patients with advanced stages who have lost the opportunity of surgical treatment. To improve the therapy efficacy, it is urgent to find more excellent EC biomarkers for early diagnosis. We have summarized the present EC-related lncRNA diagnostic biomarkers in Table 3.

**TABLE 3:** EC-associated diagnostic lncRNAs.

LncRNA name	Tumor Type	Tissues/Body Fluids	Up/down	Non-tumor Samples/EC Samples	Methods	AUC	95%CI	PMIDs
HOTAIR	ESCC	Serum	Up	20/50	RT-qPCR	0.793	0.692–0.895, *p* < 0.01	28,376,832
Linc-POU3F3	ESCC	Plasma	Up	21/21	RT-qPCR	0.842	0.794–0.890, *p* < 0.001	25,608,466
HNF1A-AS1	ESCC	Plasma	Up	21/21	RT-qPCR	0.781	0.727–0.835, *p* < 0.001	25,608,466
SPRY4-IT1	ESCC	Plasma	Up	21/21	RT-qPCR	0.800	0.748–0.853, *p* < 0.001	25,608,466
MIR31HG	ESCC	Plasma	Up	53/53	RT-qPCR	0.748	0.656–0.841, *p* < 0.01	29,605,445
MIR31HG	ESCC	Tissues	Up	53/53	RT-qPCR	0.748	0.656–0.841, *p* < 0.01	29,605,445
GHET1	ESCC	Tissues	Up	55/55	RT-qPCR	0.858	0.824–0.948, *p* < 0.05	28,983,895
FOXD2-AS1	ESCC	Tissues	Up	147/147	RT-qPCR	0.619	0.536–0.698	29,286,915
FOXD2-AS1	ESCC	Tissues	Up	147/147	RT-qPCR	0.622	0.538–0.700	29,286,915
AFAP1-AS1	ESCC	Tissues	Up	48/48	RT-qPCR	0.802	0.765–0.849, *p* < 0.001	26,756,568
SNHG1	ESCC	Serum	Up	60/60	RT-qPCR	0.850	*p* < 0.001	32,021,418
NEF	ESCC	Plasma	Down	78/78	RT-qPCR	0.9042	0.8547–0.9537, *p* < 0.05	30,402,846
HAND2-AS1	ESCC	Plasma	Down	66/66	RT-qPCR	0.9194	0.8453–0.9936, *p* < 0.0001	30,520,131
PGM5-AS1	ESCC	Plasma	Down	26/41	RT-qPCR	0.8935	0.8213–0.9658, *p* < 0.0001	31,185,143
LOC440173	ESCC	Tissues	Up	64/64	RT-qPCR	0.7205	0.6329–0.8080, *p* < 0.0001	33,079,409

ESCC, esophageal squamous cell carcinoma.

Most of the lncRNAs with diagnostic potential are located in tumor tissues. Compared with the diagnostic markers derived from tumor tissues, the markers in peripheral blood are easy to be obtained and non-invasive, which are more suitable for early screening and early diagnosis of tumors. Tong et al. (2015) identified three stable plasma ESCC-related lncRNAs. By receiver operating characteristic curve (ROC) analysis, plasma lncRNA POU3F3 could be used as a promising biomarker for the diagnosis of ESCC. A study found serum level of lncRNA HOTAIR was increased in ESCC patients, and it was positively associated with the expression of HOTAIR in ESCC tissues. Through ROC analysis, it was detected that the serum level of HOTAIR can distinguish ESCC patients from healthy controls. The results indicated that serum HOTAIR could be a promising diagnostic indicator of ESCC (Wang et al., 2017). Small nucleolar RNA host gene 1 (SNHG1) has abnormal expression in many cancers and plays a key role in ESCC (Zhang et al., 2017; Luo et al., 2020). Luo et al. (2020) discovered that SNHG1 can promote ESCC cells proliferation, and its expression level in frozen cancer tissues is significantly related to its serum level. Combined with the results of ROC analysis, it was showed that SNHG1 can be applied as a potential diagnostic biomarker for ESCC patients. In ESCC, GAS5 is considered to be a tumor suppressor (Huang et al., 2018). GAS5 serum level in EC patients was lower compared within healthy controls, and it was decreased with the primary tumor stage (T stage) increasing. Combined with the results of AUC analysis, serum GAS5 may have diagnostic values for EC (Wang et al., 2018). LncRNA PGM5 antisense RNA 1 (PGM5-AS1) was markedly decreased in ESCC cell lines, plasma and tissues. And plasma PGM5-AS1 level was promising in diagnosis of ESCC (Zhihua et al., 2019).

In summary, because of the characteristics and important functions, lncRNAs have important significance and great potential for EC diagnosis.

### 4.2 LncRNAs in Esophageal Cancer Prognosis

Recent studies have found that several lncRNAs have characteristic prognostic functions in EC (Yang et al., 2019b). Owing to the important roles in tumorigenesis and progression, combined with the stability and specificity, lncRNAs have been considered as promising biomarkers for evaluating the prognosis of EC patients, such as HOTAIR, MALAT1, non-coding RNA activated by DNA damage (NORAD), LincRNA-uc002yug.2, and long non-coding RNA activated by transforming growth factor-β (lncRNA-ATB) (Wu et al., 2014; Khorkova et al., 2015). We summarized the lncRNA prognostic biomarkers for EC in Table 4.

**TABLE 4:** EC-associated prognosis lncRNAs.

LncRNA name	High Expression	Prognosis Event	Multivariate Cox Model		Univariate Cox Model		Sample size	PMIDs
			HR (95 CI%)	*P*	HR (95 CI%)	*P*		
HOTAIR	Unfavorable	OS	NA	NA	1.913 (1.06–3.997)	*p* = 0.0334	100	24,022,190
HOTAIR	Unfavorable	OS	3.16 (1.53–6.52)	*p* = 0.002	NA	*p* < 0.001	137	24,118,380
HOTAIR	Unfavorable	MFS	4.47 (1.99–10.06)	*p* < 0.001	NA	*p* < 0.001	137	24,118,380
HOTAIR	Unfavorable	OS	2.402 (1.348–4.281)	*p* = 0.003	NA	*p* = 0.003	78	24,151,120
SPRY4-IT1	Unfavorable	OS	2.049 (1.042–4.032)	*p* = 0.038	NA	*p* = 0.016	82	24,810,925
LincRNA-uc002yug.2	Unfavorable	OS	2.57 (1.31–5.03)	*p* = 0.0021	2.39 (1.25–4.60)	*p* = 0.0062	358	25,486,427
PCAT-1	Unfavorable	OS	1.036 (1.008–1.064)	*p* = 0.011	NA	*p* = 0.001	104	25,731,728
LOC285194	Favorable	OS	0.388 (0.210–0.715)	*p* = 0.002	NA	*p* < 0.001	142	25,169,763
LOC285194	Favorable	DFS	0.341 (0.193–0.602)	*p* = 0.001	NA	*p* < 0.001	142	25,169,763
MALAT1	Unfavorable	DFS	1.76 (0.97–3.21)	*p* = 0.06	1.82 (1.01–3.27)	*p* = 0.047	77	26,406,400
MALAT1	Unfavorable	OS	1.81 (0.97–3.41)	*p* = 0.06	1.89 (1.02–3.50)	*p* = 0.043	77	26,406,400
MALAT1	Unfavorable	OS	6.638 (2.948–14.947)	*p* = 0.000	NA	*p* < 0.001	133	27,470,544
NORAD	Unfavorable	OS	3.42 (1.75–6.71)	*p* = 0.001	4.07 (2.09–7.93)	*p* = 0.000	106	28,482,344
ATB	Unfavorable	OS	1.69 (1.07–2.66)	*p* = 0.023	1.76 (1.22–2.76)	*p* = 0.014	150	28,640,252
MIR31HG	Favorable	OS	2.231 (1.118–3.899)^*^	*p* = 0.007	2.893 (1.441–4.346)^*^	*p* = 0.005	185	28,975,978
TTN-AS1	Unfavorable	OS	2.73 (1.27–4.58)	*p* = 0.002	2.54 (1.35–4.89)	*p* = 0.005	148	29,101,304
FOXD2-AS1	Unfavorable	OS	1.66 (1.04–2.64)	*p* = 0.033	1.94 (1.22–3.06)	*p* = 0.005	147	29,286,915
FOXD2-AS1	Unfavorable	DFS	2.68 (1.49–4.82)	*p* = 0.001	2.71 (1.53–4.80)	*p* = 0.000	147	29,286,915
NKILA	Favorable	OS	0.59 (0.36–0.95)	*p* = 0.029	0.42 (0.26–0.66)	*p* = 0.000	137	29,348,395
LINC01503	Unfavorable	OS	1.967 (1.112–3.479)	*p* = 0.020	1.891 (1.074–3.327)	*p* = 0.027	113	29,454,790
LINC01503	Unfavorable	DFS	1.583 (0.975–2.570)	*p* = 0.063	1.753 (0.958–2.518)	*p* = 0.035	113	29,454,790
LINC01133	Favorable	OS	0.500 (0.307–0.812)	*p* = 0.005	0.381 (0.243–0.599)	*p* < 0.001	149	30,007,982
LINC01296	Unfavorable	OS	2.893 (1.253–5.563)	*p* = 0.004	3.783 (1.669–7.693)	*p* = 0.001	221	30,058,683
LINC01296	Unfavorable	DFS	3.263 (1.193–6.763)	*p* = 0.003	4.213 (1.389–8.784)	*p* = 0.001	221	30,058,683
AK001796	Unfavorable	OS	3.347 (1.423–5.457)	*p* = 0.005	NA	*p* = 0.010	175	30,657,559
AK001796	Unfavorable	DFS	3.568 (1.537–5.778)	*p* = 0.003	NA	*p* = 0.001	175	30,657,559
MEG3	Favorable	OS	2.638 (1.052–6.612)^*^	*p* = 0.039	5.737 (2.653–12.404)^*^	*p* < 0.001	58	30,990,378
MEG3	Favorable	DFS	2.765 (1.045–7.315)^*^	*p* = 0.040	6.937 (2.892–16.641)^*^	*p* < 0.001	58	30,990,378
LOC100133669	Unfavorable	OS	2.009 (1.340–3.010)	*p* = 0.001	NA	NA	155	32,130,753
LEF1-AS1	Unfavorable	OS	2.942 (1.169–4.156)	*p* = 0.014	3.162 (1.225–4.654)	*p* = 0.008	185	31,599,448
LEF1-AS1	Unfavorable	DFS	2.856 (1.123–4.327)	*p* = 0.017	3.056 (1.218–4.664)	*p* = 0.008	185	31,599,448
SBF2-AS1	Unfavorable	CS	NA	NA	1.31 (1.090–1.568)	*p* = 0.016	60	29,552,140
ZEB1-AS1	Unfavorable	OS	2.371 (1.284–6.115)	*p* = 0.013	NA	*p* = 0.03	87	26,617,942
ZEB1-AS1	Unfavorable	DFS	2.695 (1.379–8.352)	*p* = 0.007	NA	*p <* 0.05	87	26,617,942
FMR1-AS1	Unfavorable	OS	NA	NA	1.618 (1.117–2.345)	*p* = 0.009	206	30,736,860
FMR1-AS1	Unfavorable	OS	NA	NA	1.768 (1.189–2.631)	*p* = 0.0031	188	30,736,860
TUG1	Unfavorable	OS	1.403 (1.012–1.946)	*p* = 0.042	1.640 (1.194–2.255)	*p* = 0.002	218	27,329,359
UCA1	Unfavorable	OS	2.627 (1.416–5.874)	*p* < 0.001	2.931 (1.72–6.214)	*p* = 0.006	90	25,550,835
PVT1	Unfavorable	OS	2.75 (1.35–5.59)	*p* = 0.05	3.65 (1.87–7.14)	*p <* 0.001	104	28,404,954
ANRIL	Unfavorable	OS	0.271 (0.128–0.574)^*^	*p* = 0.001	0.292 (0.111–0.768)^*^	*p* = 0.013	50	30,610,814
ANRIL	Unfavorable	DFS	0.335 (0.161–0.699)^*^	*p* = 0.004	0.321 (0.126–0.814)^*^	*p* = 0.017	50	30,610,814
XIST	Unfavorable	OS	2.40 (1.44–4.01)	*p* = 0.001	2.06 (1.25–3.40)	*p* = 0.005	12	29,100,288
AFAP1-AS1	Unfavorable	OS	1.888 (1.223–2.915)	*p* = 0.004	2.665 (1.838–3.865)	*p* < 0.001	162	26,756,568
AFAP1-AS1	Unfavorable	PFS	1.626 (1.057–2.501)	*p* = 0.027	2.242 (1.545–3.255)	*p* < 0.001	162	26,756,568
SNHG1	Unfavorable	OS	3.432 (1.951–5.064)	*p* = 0.006	6.851 (4.356–11.867)	*p* < 0.001	42	32,021,418
SNHG1	Unfavorable	DFS	3.016 (1.294–4.645)	*p* = 0.009	6.054 (3.284–9.852)	*p* < 0.001	42	32,021,418
CCAT1	Unfavorable	OS	1.044 (1.023–1.066)	*p* < 0.001	1.053 (1.034–1.072)	*p* < 0.001	90	27,956,498
H19	Unfavorable	OS	NA	NA	2 (1.22–3.28)	*p* = 0.0052	234	31,417,277
LOC440173	Unfavorable	OS	2.375 (1.043–5.405)	*p* = 0.039	2.608 (1.205–5.643)	*p* = 0.015	64	33,079,409
LOC440173	Unfavorable	RFS	2.261 (1.005–5.090)	*p* = 0.049	2.510 (1.161–5.427)	*p =* 0.019	64	33,079,409
LEF1-AS1	Unfavorable	OS	2.942 (1.169–4.156)	*p =* 0.014	3.162 (1.225–4.654)	*p =* 0.008	185	31,599,448
LEF1-AS1	Unfavorable	DFS	2.856 (1.123–4.327)	*p =* 0.017	3.056 (1.218–4.664)	*p =* 0.008	185	31,599,448

NA, Not Available; OS, Overall Survival; DFS, Disease-Free Survival; PFS, Progression-Free Survival; CS, Cumulative Survival; MFS, Metastasis-Free Survival; RFS, Recurrence-Free Survival.

* The HR value was calculated by high expression of lncRNA as reference.

As previously mentioned, HOTAIR is an important oncogenic lncRNA and has great potential for EC diagnosis. At the same time, it is also an important prognostic biomarker for EC. Kaplan–Meier survival curves showed, compared with patients with low HOTAIR expression, patients with high HOTAIR expression had noticeably shorter survival times (HR value >1). The results indicate that HOTAIR is an unfavorable factor for the prognosis of EC and can be used as a prognostic biomarker for EC (Ge et al., 2013; Li et al., 2013). LncRNA MALAT1 is located on chromosome 11q13.1 and its expression is conserved in multiple species (Ji et al., 2003; Huang et al., 2016). MALAT1 is involved in multiple biological functions and overexpressed in many cancers, such as liver cancer, lung cancer, renal cancer, gastric cancer (Ji et al., 2003; Su et al., 2021). MALAT1 acts on proliferation, metastasis, cell cycle and drug resistance in these cancer cells (Su et al., 2021). Compared with the expression in adjacent non-carcinoma tissues, MALAT1 expression is higher in EC tissues and correlates with unfavorable prognosis (Wang et al., 2019b; Syllaios et al., 2021). MALAT1 promotes EC cell proliferation, migration and invasion (Wang et al., 2019b; Syllaios et al., 2021). According to the multivariate analysis of prognostic factors (HR,6.638, 95% CI,2.948–14.947), MALAT1 was significantly related to the prognosis of EC patients and could be applied as an independent factor to EC prognosis (Huang et al., 2016). NORAD, a 5300 nt lncRNA and annotated in RefSeq as LINC00657, is a highly conserved lncRNA. It takes a primary part in regulating both ploidy and chromosomal stability in diploid cells. And the loss of NORAD function can result in chromosomal instability (Lee et al., 2016). Importantly, lncRNA NORAD is overexpressed in many cancers, including breast cancer, gastric cancer, bladder cancer, liver cancer, and so on (Soghli et al., 2021). Studies showed that NORAD could act as a ceRNA (Yang et al., 2019c). A meta-analysis reported that higher NORAD expression was significantly related with poorer overall survival in cancers (Wang et al., 2021a). And its expression level was appreciably higher in ESCC tissues than that in adjacent normal tissues. The multivariate analysis indicated that NORAD was an independent predictor of ESCC overall survival, which showed that NORAD is a potential ESCC prognostic marker (Wu et al., 2017).

In addition, many lncRNAs, such as LincRNA-uc002yug.2, ATB, are shown to be potential prognostic biomarkers for EC by the Multivariate Cox regression model (Table 4). Whether these lncRNAs can be further applied in clinic needs further verification, and the combined application of multiple lncRNAs is an important direction for future research.

### 4.3 LncRNAs in Esophageal Cancer Therapy

Therapy resistance leads to poor treatment efficacy and high recurrence rate of cancers to result in dismal prognosis. Resistance to cancer therapy ordinarily stems from deregulation of signaling pathways. LncRNAs are involved in many these pathways (Askarian-Amiri et al., 2016; Majidinia and Yousefi, 2016; Borkiewicz et al., 2021). For example, exogenous lncRNA UCA1 increased the invasiveness of cancer cells and worked in cisplatin resistance to bladder cancer therapy (Wang et al., 2008). LncRNA HOTAIR overexpression increased breast cancer cell proliferation and contributed to tamoxifen resistance in breast cancer (Xue et al., 2016). We summarized the lncRNAs involved in resistance of EC therapy (Table 5).

**TABLE 5:** LncRNAs and drugs resistance/radioresistance in EC.

LncRNAs	Cancer Type	Up/down	Related Therapeutics Resistance	Target/Pathway	PMIDs
PCAT-1	EC	Up	Cisplatin	NA	29,314,203
TUSC7	ESCC	Down	Cisplatin and 5-Fu	DESC1/EGFR/AKT pathway	29,530,057
NMR	ESCC	Up	Cisplatin and paclitaxel	NA	29,763,634
LINC00261	ESCC	Down	5-Fu	DYPD	30,226,808
Linc-VLDLR	ESCC	Up	Adriamycin	ABCG2	30,606,658
LINC00337	ESCC	Up	Cisplatin	TPX2/E2F4	32,239,565
TUG1	ESCC	Up	Radioresistance	miR-144-3p/MET/EGFR/AKT pathway	31,918,742
TUG1	ESCC	Up	Cisplatin	Nrf2	31,287,493
TUG1	ESCC	Up	Cisplatin	PDCD4	30,519,392
TUG1	ESCC	UP	Platinum combined with 5-Fu or paclitaxel	NA	27,329,359
MALAT1	ESCC	Up	Radioresistance	Cks1	27,935,117
FOXD2-AS1	ESCC	Up	Cisplatin	miR-195/Akt/mTOR pathway	31,558,183
NMR	ESCC	Up	Cisplatin	NSUN2/BPTF/MMP3 axis, and MMP10	29,763,634
TP73-AS1	ESCC	Up	5-Fu and cisplatin	BDH2	26,799,587
CCAT1	ESCC	Up	Cisplatin	CDK4	31,544,294
CCAT2	EC	Up	Radioresistance	Bax/Bcl2	31,789,385
DDX11-AS1	EC	Up	Paclitaxel	TAF1	31,720,085
H19	ESCC	Up	Radiotherapy	WNT1 via miR-22-3p	31,417,277
AFAP1-AS1	ESCC	Up	Cisplatin	NA	26,756,568

EC, esophageal cancer; ESCC, esophageal squamous cell carcinoma; NA, Not Available; 5-Fu, 5-fluo-rouracil.

MALAT1 is a potential prognostic biomarker for EC. Knockdown of MALAT1 could enhance the radiosensitivity and chemosensitivity of ESCC cells (Yao et al., 2019). And MALAT1 overexpression inhibited the viability decrease and apoptosis increase of EC cells which were induced by irradiation (Li et al., 2017).

H19 is an oncogenic lncRNA and takes part in the tumorigenesis and progression of EC (Huang et al., 2015). Radioresistance is a main factor limiting the efficacy of radiotherapy for EC. H19 was upregulated in an ESCC radioresistant cell line. And H19 knockdown downregulated Wnt1 through upregulating miR-22-3p expression, then resulted in the inhibition of radioresistance, proliferation and migration in radioresistant ESCC cell (Luo et al., 2019).

Prostate cancer associated transcripts 1 (PCAT-1) was first discovered in patients with prostate cancer by transcript sequencing and was identified as a transcriptional repressor (Prensner et al., 2011). LncRNA PCAT-1 is up-regulated and could play an oncogenic role in multiple cancers, such as prostate cancer, ESCC, gastric cancer, ovarian cancer (Prensner et al., 2011; Shi et al., 2015; Bi et al., 2017; Ding et al., 2019). PCAT-1 expression is remarkably increased in ESCC tissues, which is significantly related to tumor invasion (Shi et al., 2015; Razavi and Ghorbian, 2019). And it was found that PCAT-1 facilitated ESCC progression by PCAT-1/miR-508-3p/ANXA10 axis in cell experiments (Zang et al., 2019). When PCAT-1 was inhibited, the chemosensitivity of EC to cisplatin was increased (Zhen et al., 2018).

Taurine up-regulated gene 1 (TUG1) is a lncRNA which is abnormally expressed in various tumors (Da et al., 2021). It was found that TUG1 could function as ceRNA to specifically sponging microRNAs to regulate gene expression and was involved in oncogenesis and development of many malignant tumors, such as gastric cancer (Ren et al., 2017), osteosarcoma (Sheng and Li, 2019). And TUG1 could regulate resistance and sensitivity of some cancers to chemotherapeutic drugs, such as bladder cancer (Yu et al., 2019), cervical cancer (Wei et al., 2019) and EC. TUG1, as an oncogenic lncRNA in EC, is significantly upregulated in EC and promotes EC development (Jin et al., 2020; Tang et al., 2020; Zong et al., 2020). TUG1 is also related to radiotherapy resistance and chemotherapy resistance of ESCC. Patients with high TUG1 expression displayed more resistance to chemotherapy (platinum-based chemotherapy combined with paclitaxel or 5-fluo-rouracil) compared with low TUG1 expression group (Jiang et al., 2016). Mechanically, TUG1 can promote cisplatin resistance in ESCC through upregulating Nrf2 or epigenetically suppressing PDCD4 expression *via* EZH2 (Xu et al., 2018a; Zhang et al., 2019c). In addition, TUG1 can increase radiotherapy resistance of ESCC by reducing miR-144-3p and regulating MET/EGFR/AKT axis (Wang et al., 2020).

Notably, lncRNA POU3F3 was reported to promote ESCC cell proliferation and cisplatin resistance through exosomal POU3F3-induced transformation of normal fibroblasts to cancer-associated fibroblasts in ESCC (Tong et al., 2020). However, studies about exosomal lncRNAs and chemoresistance are relatively scarce, especially those about the association between lncRNAs and multidrug resistance in EC.

In summary, recent advances have found that many lncRNAs participate in the drug resistance of EC. However, the exact role of lncRNAs is unclear in the entire regulatory network of drug resistance, and exploring the underlying mechanism will help us to reverse drug resistance of EC via using lncRNA-targeting strategy.

## 5 Conclusion and Future Perspectives

With the high-speed development of high-throughput sequencing technology, Genome Map and bioinformatics, a great deal of lncRNAs have been discovered. LncRNA is involved in various processes such as epigenetic regulation, chromatin remodeling and gene expression regulation (Shi et al., 2013; Beermann et al., 2016; Quinn and Chang, 2016). Many human diseases, including cancers, are related to the dysregulation of lncRNA (Bhan et al., 2017; Chi et al., 2019). Increasing evidence indicates that lncRNAs are involved in regulating the carcinogenesis and progression of EC. Importantly, these dyregulated lncRNAs may be useful for the early diagnosis, prognosis, and treatment of EC.

Nowadays, the researches of lncRNA mainly focus on several aspects: identification and classification of lncRNA; the interaction between lncRNA and other factors; the roles of lncRNA in major diseases; the potential of lncRNA as biomarkers and drug targets of diseases. The role of lncRNA in cancers is a hotspot. In this paper, we mainly summarized the biological functions and molecular mechanisms of lncRNAs. At the same time, we reviewed the main oncogenic lncRNAs and suppressive lncRNAs in EC, and lncRNAs related to EC diagnosis, treatment, and prognosis. The previous studies have shown that lncRNAs play important roles in the occurrence and development of EC and hold much promise as novel biomarkers and therapeutic targets for EC.

However, the research on lncRNAs in EC is still in initial phase and faced with a number of challenges especially in clinical application. Moreover, several novel mechanisms of lncRNAs in EC should be focused on in the future. Currently, despite the dysregulated lncRNAs seem to be important in the tumorigenesis or progression, there are still some questions required to be answered by further studies before their clinical translation. First, although multiple screening wet methods (microarrays, next-generation sequencing and qRT-PCR) have been used to identify the cancer-associated lncRNAs, more effective bioinformatics tools need to be developed. OSescc for identifying the prognosis-related genes in ESCC patients (Wang et al., 2019d), have been developed in recent years, however available clinical lncRNA testing method has not been seen to now. Second, how do lncRNAs co-regulate with other functional molecules (such as miRNAs, circRNAs, proteins) in the pathogenesis of EC? For example, the disordered regulatory networks of lncRNAs-miRNAs-mRNAs can induce the occurrence and development of EC (Karreth and Pandolfi, 2013). Third, the small peptides encoded by ncRNAs have been recurrently reported (Wang et al., 2019c). It will be very interesting to explore whether these lncRNAs encode small peptides, which is the direct regulator for malignant behaviors of EC.

In addition, increasing evidences showed that EC-derived exosomes may be useful for the early detection of EC. RNA sequencing revealed that dysregulated exosomal lncRNAs are associated with early-stage ESCC (Tian et al., 2020). Some lncRNA-based signature shows the higher efficiency of the prognosis of EC. Liu et al. (2020b) reported a toll-like receptor (TLR)-related four-lncRNA signature as a prognostic biomarker in EC. Moreover, several effective machine learning methods have been developed to identify and predict lncRNAs in various organisms (Xu et al., 2021c). For instance, Liu et al. (2022) constructed an immune-associated lncRNA signature to predict the clinical outcome of colorectal cancer patients based on machine learning-based methods. Zhang et al. (2018c) constructed the CRlncRC, a machine learning-based method for identifying the cancer-related lncRNAs. Therefore, the application of machine learning-based lncRNAs in the diagnosis of EC will be interesting.

In brief, lncRNAs might be promising biomarkers or therapeutic targets for clinical application for EC after in-depth basic and clinical investigations.
